# Insight into the Storage Mechanism of Sandwich-Like Molybdenum Disulphide/Carbon Nanofibers Composite in Aluminum-Ion Batteries

**DOI:** 10.3390/nano14050442

**Published:** 2024-02-28

**Authors:** Xiaobing Wang, Ruiyuan Zhuang, Xinyi Liu, Mingxuan Hu, Panfeng Shen, Jintao Luo, Jianhong Yang, Jianchun Wu

**Affiliations:** 1 School of Advanced Materials Engineering, Jiaxing Nanhu University, Jiaxing 314000, China; 2 School of Mechanical and Electrical Engineering, Jiaxing Nanhu University, Jiaxing 314000, China; 3 School of Materials Science and Engineering, Jiangsu University, Zhenjiang 212013, China; 4 Institute of Nuclear Science and Technology, Sichuan University, Chengdu 610064, China

**Keywords:** aluminum-ion batteries, electrochemical behavior, energy storage mechanism, MoS_2_/CNFs, composite materials

## Abstract

Aluminum-ion batteries (AIBs) have become a research hotspot in the field of energy storage due to their high energy density, safety, environmental friendliness, and low cost. However, the actual capacity of AIBs is much lower than the theoretical specific capacity, and their cycling stability is poor. The exploration of energy storage mechanisms may help in the design of stable electrode materials, thereby contributing to improving performance. In this work, molybdenum disulfide (MoS_2_) was selected as the host material for AIBs, and carbon nanofibers (CNFs) were used as the substrate to prepare a molybdenum disulfide/carbon nanofibers (MoS_2_/CNFs) electrode, exhibiting a residual reversible capacity of 53 mAh g^−1^ at 100 mA g^−1^ after 260 cycles. The energy storage mechanism was understood through a combination of electrochemical characterization and first-principles calculations. The purpose of this study is to investigate the diffusion behavior of ions in different channels in the host material and its potential energy storage mechanism. The computational analysis and experimental results indicate that the electrochemical behavior of the battery is determined by the ion transport mechanism between MoS_2_ layers. The insertion of ions leads to lattice distortion in the host material, significantly impacting its initial stability. CNFs, serving as a support material, not only reduce the agglomeration of MoS_2_ grown on its surface, but also effectively alleviate the volume expansion caused by the host material during charging and discharging cycles.

## 1. Introduction

In response to global climate change, the construction of a green, low-carbon, and clean energy system with renewable energy as the mainstay is the energy strategy of the world. However, the intermittency and randomness of renewable energy have brought great challenges to maintaining the balance of power and electricity in power systems. Addressing the imbalance between renewable energy generation and electricity load demands the utilization of energy storage technology [1,2]. Therefore, large-scale energy storage technology is perceived as a pivotal strategy to facilitate the widespread adoption of renewable energy, serving as a strategic underpinning for the future transformation of energy structures and shifts in power production and consumption patterns. At present, lithium-ion batteries (LIBs) are facing challenges, including the scarcity of lithium resources and safety concerns, hindering their widespread adoption in the energy storage field [3,4]. Consequently, the development of cost-effective and high-safety secondary batteries has garnered widespread attention from society.

Aluminum-ion batteries (AIBs) have the advantages of abundant aluminum resources, high safety, and high theoretical volumetric capacity (8040 mAh cm^−3^). Compared with traditional LIBs, AIBs have multiple electrons participating in the electrode reaction during the charging and discharging processes, resulting in a high theoretical specific capacity, making AIBs a growing research hotspot in the field of energy storage [5,6]. Since Holleck used aluminum metal as the anode; metal chloride as the cathode; and molten salt electrolyte consisting of AlCl_3_, KCl, and NaCl to form aluminum batteries, research on non-aqueous AIBs has begun to receive attention from researchers [7]. However, due to the high electrode potential of aluminum and the large ionic radius involved in the electrode reaction, AIBs have lower energy density and insufficient electrochemical performance to meet practical demands. In 2015, Lin introduced a model of AIBs utilizing a novel ionic liquid (AlCl_3_/[EMIm]Cl) as the electrolyte, marking a significant breakthrough in the field of AIBs [8]. Subsequently, various materials have been integrated into the cathode materials of non-aqueous AIBs, including metal oxides (such as MnO_2_ and V_2_O_5_) [9,10], transition metal chalcogenides (such as CuS_2_, VS_2_, and Co_9_S_8_) [11,12,13], carbon-based materials (such as graphene, graphitic carbon, and graphitic foam) [14,15,16], and other materials (such as MXenes and conducting polymers) [17,18]. Among them, carbon-based materials are one of the most used in AIBs, with advantages such as high discharge voltage and good cycle stability. However, in the storage mechanism of carbon-based materials, the guest ion is AlCl_4_^−^, whose theoretical specific capacity is only one-third of Al^3+^ under the same amount of ion insertion, resulting in insufficient discharge capacity (approximately 70 mAh g^−1^). Transition metal oxides have gained significant attention for their higher discharge specific capacity compared to carbon materials. Nevertheless, current research on transition metal oxides mainly focuses on vanadium-based oxides, mainly due to the high surface charge density of Al^3+^, coupled with the high transmission barrier of multivalent ions in oxide crystal structures, which leads to strong coulombic interactions between Al^3+^ and the material lattice, hindering its interlattice diffusion [19,20]. Compared to other cathode materials (such as carbon-based materials, transition metal chalcogenides, et al.), transition metal chalcogenides can facilitate the exchange of three electrons during aluminum storage, thereby exhibiting a high theoretical specific capacity. Additionally, transition metal chalcogenides possess advantages such as wide interlayer spacing, low electronegativity, and abundant resources. Moreover, the coulombic interaction between Al^3+^ and transition metal chalcogenides is comparatively weaker than that observed with metal oxides. Consequently, they are considered highly promising cathode materials for AIBs [21].

Molybdenum disulfide (MoS_2_) is a sulfide with a sandwich-like layered structure, similar to graphene, but with a distinct stacking sequence. Each MoS_2_ layer constitutes a three-dimensional structure via strong S-Mo-S ionic/covalent bonds, and each adjacent layer of MoS_2_ is separated by van der Waals forces, forming a spacing of 0.62 nm. This spacing is notably larger than the interlayer spacing of graphite (0.335 nm) [22,23]. The weaker van der Waals forces between the layers of MoS_2_ facilitate the rapid diffusion of ions without significant changes in volume, making MoS_2_ an ideal host material for electrochemical intercalation and deintercalation. Indeed, MoS_2_ has been reported as an electrode material in LIBs, sodium-ion batteries (SIBs), zinc-ion batteries (ZIBs), and AIBs [24,25,26,27,28,29].

Li applied petal-shaped MoS_2_ microspheres to AIBs for the first time. Electrochemical testing results revealed that the initial discharge specific capacity was 253.6 mAh·g^−1^ at a current density of 20 mA·g^−1^, and the discharge specific capacity after 100 cycles was 66.7 mAh·g^−1^ at a current density of 40 mA·g^−1^. The large ionic radius involved in the electrode reaction during charging and discharging resulted in a low discharge specific capacity and poor cycle stability [27]. Researchers have made structural designs and optimized the properties to improve their inherent defects and enhance their electrochemical performance [28,29]. Subsequently, Luo prepared MoS_2_-RGO composite electrode materials using graphene as a carrier, and Lu designed MoS_2_@CNFs composite electrodes using carbon nanotubes as a carrier [28,29]. The introduction of graphene and carbon nanotubes in the composite electrode effectively improved the electrical conductivity of the electrode material, increased the contact area between the electrode material and the electrolyte, and then effectively improved the electrochemical performance of the electrode. Therefore, in addition to designing the electrode structure reasonably, it is crucial to study the energy storage mechanism of MoS_2_, as it has been demonstrated that MoS_2_ is a suitable material for AIBs. Understanding the energy storage mechanism may help to successfully design stable electrode materials and optimize their performance.

Here, MoS_2_/CNFs composites were synthesized using a simple electrospinning and subsequent hydrothermal method, with layered MoS_2_ evenly distributed on the surface of the CNFs. Serving as a binder-free electrode for AIBs, the uniform nanosheet and layered structure of MoS_2_/CNFs ensures complete contact with the electrolyte, improving its conductivity, thereby promoting the transport of Al^3+^ and the cycling performance. Additionally, the carbon substrate not only prevents the agglomeration of MoS_2_, but also alleviates the volume expansion effects of the electrode during charging and discharging. As a result, the prepared AIBs exhibit good cycling stability, delivering a first discharge capacity of approximately 180 mAh g^−1^ at 100 mA g^−1^, and maintaining their capacity at about 53 mAh g^−1^ after 260 cycles. Ex-situ XPS and XRD characterization were conducted to gain a deeper understanding of the energy storage mechanism of the MoS_2_-based cathode. Consequently, the deintercalation energy of ions in different channels of MoS_2_ was calculated, and the potential ion deintercalation sites and energy storage mechanism were inferred based on the magnitude of the deintercalation energy. Specifically, the layer distance of MoS_2_ matches the size of Al^3+^, and Al^3+^ is inserted into MoS_2_ to generate AlxMoS_2_.

## 2. Materials and Methods

### 2.1. Synthesis of Binder-Free MoS_2_/CNFs Cathode

#### 2.1.1. Electrospinning

**Figure 1:**
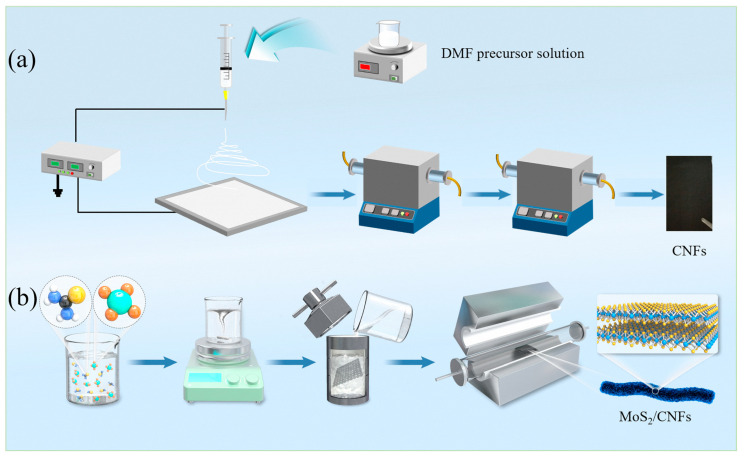
Schematic illustration of the preparation process: (**a**) CNFs; (**b**) MoS_2_/CNFs.

All reagents were used without further purification. A weight of 3 g of PAN (PAN, average Mw = 150,000, ALDRICH, Shanghai, China) was dissolved in 27 mL of N,N-dimethyformamide (DMF, 99.9%, innochem) solution and vigorously stirred for 12 h until the solution became uniform and clear, and then it was poured into a needle tube. After that, the plastic needle tube was placed on the spinning machine injection pump for electrospinning. The parameters of the spinning process were: high-voltage power supply voltage of 15 kV, push rate of 0.5 mL h^−1^, and needle diameter of Φ 18. After electrospinning, the fiber precursor was collected for thermal treatment. Under air atmosphere, the fibers were pre-treated at 280 °C with a temperature rise rate of 1 °C min^−1^. The fiber obtained after pre-treatment was a brownish black. Finally, the pre-treated fibers were further carbonized, which needs to be carried out in Ar atmosphere at 800 °C with a temperature rise rate of 5 °C min^−1^, as shown in Figure 1a.

#### 2.1.2. Hydrothermal Method

Initially, 2 mmol (NH_4_)_6_Mo_7_O_24_ (99.95%, Sinopharm Chemical Reagent Co., Ltd., Shanghai, China) and 10 mmol CH_4_N_2_S (99.95%, Sinopharm Chemical Reagent Co., Ltd., Shanghai, China) were introduced into 80 mL of deionized water, followed by stirring at a speed of 600 r min^−1^ for a duration of 2 h until complete dissolution was achieved. The solution was then transferred to a 100 mL PTFE-lined autoclave. Subsequently, the CNFs prepared above were added to the solution. The PTFE-lined autoclave was reacted at 200 °C for 24 h with a heating rate of 2 °C min^−1^. After completion of the reaction, the sample was thoroughly washed and dried overnight at 60 °C in a vacuum oven with a heating rate of 1 °C min^−1^. Finally, the hybrid composite materials were calcined at 350 °C for 2 h with a heating rate of 2 °C min^−1^ under a nitrogen atmosphere to yield the desired MoS_2_/CNFs composite (depicted in Figure 1b). The synthesis process for pure MoS_2_ nanomaterials is comparable to the preparation method for MoS_2_/CNFs, except that pure carbon fibers are not employed as the matrix material during the synthesis process.

### 2.2. Structural and Chemical Characterization

X-ray diffraction (XRD, SmartLab 9kw, Shanghai, China) analysis was employed for the phase characterization of the synthesized sample, operating within an angle range of 5–90° and a scan rate of 10° min^−1^. The Thermo Fisher Nexsa (Waltham, MA, USA) instrument was utilized to analyze the surface element composition and valence states. The analysis chamber was operated at a vacuum level of approximately 5 × 10^−9^ mbar, utilizing a monochromatic Al-Kα X-ray source with an energy of 1486.6 eV and a voltage of 12 KV. The Thermo Fisher DXR instrument was employed to depict the molecular vibration and rotational energy level structure of the synthesized material, operating with a 523 nm laser and a power of 1 mW. To acquire a comprehensive understanding of the physical and chemical attributes, such as morphology, composition, and microstructure, the scanning electron microscopy (JSM-7800F, JEM-2100, JEOL, Tokyo, China) was researched.

### 2.3. Electrochemical Measurements

Considering the high corrosiveness of the ionic liquid electrolyte employed in the experiment towards stainless steel, soft-pack batteries were assembled for testing in this work. The batteries consist of binder-free electrodes made of MoS_2_/CNFs, glass fiber separators, aluminum foils, and ionic liquid electrolytes. Specifically, the ionic liquid electrolyte was prepared by combining AlCl_3_ (99.999%, Sigma Aldrich, St. Louis, MO, USA) and 1-ethyl-3-methylimidazolium chloride ([EMIm]Cl, 98%, Aladdin, Shanghai, China) in a molar ratio of 1:1.3. After assembly, the batteries were allowed to rest for 6 h to ensure the electrolyte fully contacted the electrode materials. All constant current charge–discharge tests were conducted on the Neware electrochemical testing system, with a test voltage range of 0.1–1.8 V (relative to Al/AlCl_4_^−^). In order to study the electrochemical activity and reaction process of the electrodes, CV tests were performed on the PARSTAT MCEIS instrument of the Princeton Electrochemical Workstation (AMETEK, Middleboro, MA, USA).

### 2.4. Computational Details

The simulations were conducted by the first-principles calculations based on density functional theory (DFT). For the computational simulation of MoS_2_, the spatial symmetry group is P63/mcc (194). The computational model employed a 4 × 4 × 1 MoS_2_ supercell structure, with a supercell size of 12.76 × 12.76 × 18.88 Å3. Considering the influence of van der Waals interactions, the DFT-D3 method was used for vdW correction during the calculation process. Prior to calculating the Al^3+^ insertion structure, the model was fully optimized for structural optimization.

## 3. Results

**Figure 2:**
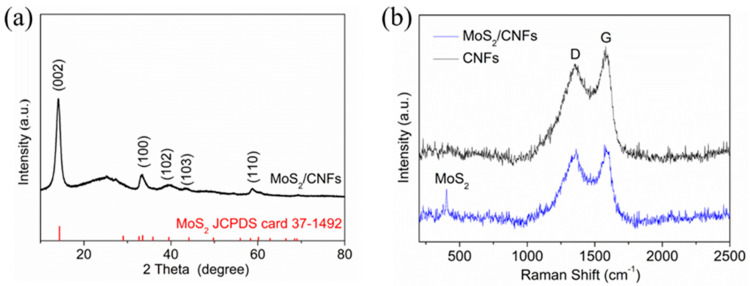
(**a**) Ex−situ XRD patterns and (**b**) Raman patterns of the MoS_2_/CNFs.

Figure 2a shows the XRD pattern of the MoS_2_/CNFs composite material. The diffraction peaks of the synthesized sample match with the (002), (100), (102), (103), and (110) crystal planes of the standard card (JCPDS No. 37-1492) for 2H-MoS_2_. From the figure, it can be seen that no impurity peaks of molybdenum or other impurities were observed, indicating that the synthesized product is pure-phase MoS_2_. The peak at 2 θ = 25 ° can be attributed to amorphous carbon. Moreover, it can be concluded that the synthesized MoS_2_ has a P63/mmc space group and belongs to the hexagonal crystal system. However, compared to the standard card, all diffraction peaks of MoS_2_/CNFs are shifted to the left by a small angle, especially the diffraction peaks of the (002) crystal plane, which is most affected by CNFs. Raman spectroscopy was conducted to further confirm the consistency of the phase and chemical composition of the synthesized materials, and the results are shown in Figure 2b. The Raman spectrum of pure CNFs only has two distinct characteristic peaks, corresponding to the D and G peaks, respectively. However, the Raman spectrum of MoS_2_/CNFs also has two additional characteristic peaks, located at 378 and 406 cm^−1^, which correspond to the E_2g_ and A_1g_ of 2H-MoS_2_ [30,31]. Therefore, through XRD and Raman characterization, MoS_2_/CNFs composite materials were successfully synthesized.

**Figure 3:**
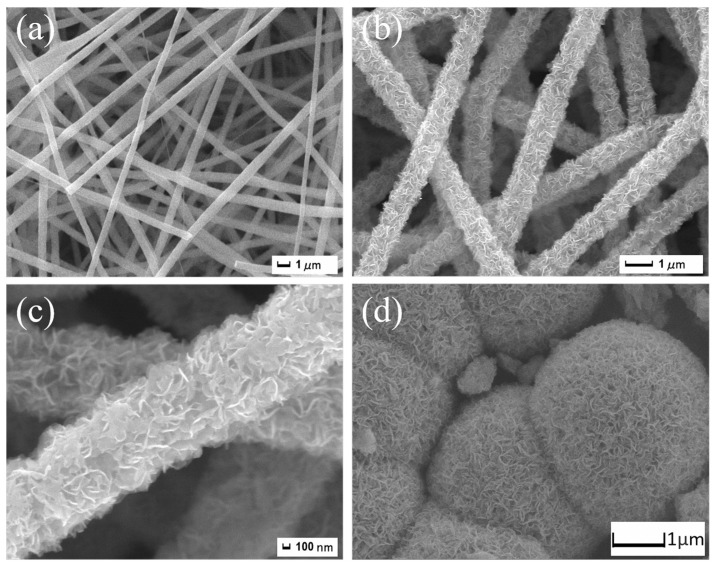
(**a**) SEM image of pure CNFs; (**b**,**c**) SEM images of MoS_2_/CNFs; (**d**) SEM image of pure MoS_2_.

To gain a better understanding of the morphology of the synthesized materials, SEM was performed. Figure 3a shows the corresponding SEM image of pure CNFs. It can be observed that the pure CNFs have a relatively smooth surface and uniform fiber diameter. Although the fibers have been calcined at high temperatures, they still maintain a long-range continuous morphology and form a three-dimensional (3D) network structure. After the hydrothermal synthesis process, the surface of the fiber becomes slightly rough, and layered MoS_2_ nanosheets are uniformly grown on the fiber surface (Figure 3b,c). Due to the strong mechanical strength of CNFs, the 3D network structure of the composite material remains well after hydrothermal reaction. In order to further demonstrate the superiority of carbon nanofibers as a matrix material, SEM characterization was also performed on pure MoS_2_. As shown in Figure 3d and Supplementary Figure S1, the MoS_2_ nanomaterials prepared by one-step hydrothermal synthesis exhibit a clustered morphology formed by nanosheets. The main reason is that during the synthesis process, due to excessive reaction time, the layered structure gradually converges to form a spherical shape. However, the MoS_2_ nanoballs are irregular and their size is large, exceeding 1 μm. Therefore, using carbon fibers as a substrate can effectively prevent the agglomeration of MoS_2_ during formation. In addition, epitaxially grown MoS_2_ nanosheets construct nanovoids on one-dimensional CNFs, which is beneficial for electrode/electrolyte interactions when used as battery electrodes.

**Figure 4:**
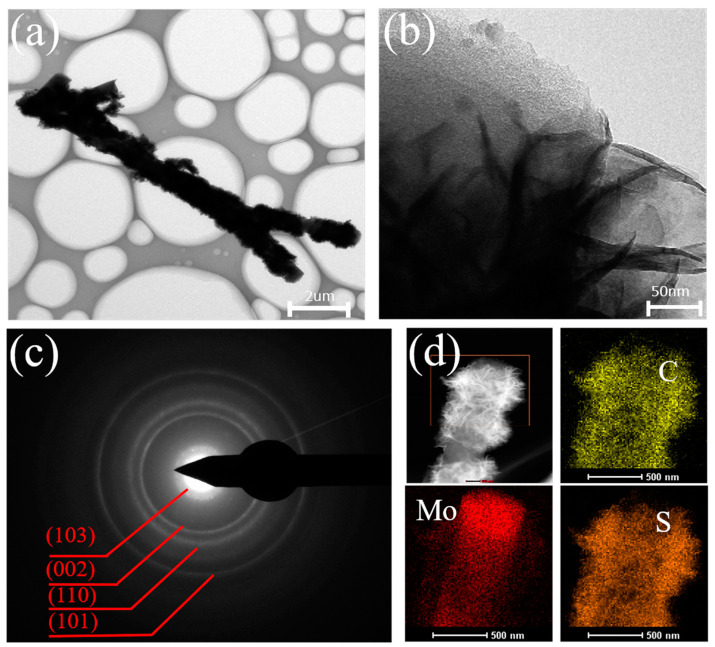
(**a**,**b**) TEM figures of MoS_2_/CNFs; (**c**) SAED patterns of MoS_2_/CNFs; (**d**) corresponding mapping patterns of MoS_2_/CNFs.

In addition, TEM characterization was studied to further analyze the morphology and microstructure of the prepared products. Figure 4a,b present TEM images of MoS_2_/CNFs at varying magnifications. The observations in Figure 4a are comparable to those from the preceding SEM images, clearly showing the uniform growth of MoS_2_ nanosheets on the fiber surface. From Figure 4b, the magnified TEM image reveals that the MoS_2_ nanosheets on the fiber surface are relatively thin and have a clearly visible layered structure with a thickness of approximately 30 nm. Compared to MoS_2_ nanoballs synthesized by a one-step hydrothermal method, small-sized MoS_2_ nanosheets are more suitable as electrode materials for batteries, as smaller sizes of active materials can effectively improve electron transfer ability and shorten ion migration distance. Furthermore, the MoS_2_ in the MoS_2_/CNFs composite material is minimally aggregated and exposes a greater number of active sites. These factors indicate that the preparation process of growing active substances on the surface of CNFs using the hydrothermal method is superior. The polycrystalline nature of MoS_2_/CNFs can be verified by a series of concentric rings in Figure 4c. The selected area electron diffraction pattern reveals that these rings correspond to the (103), (002), (110), and (101) planes of MoS_2_. Figure 4d depicts the energy dispersive X-ray spectroscopy (EDS) of MoS_2_/CNFs, revealing that the composite material comprises three elements of C, Mo, and S, and each element is evenly distributed, indicating that the MoS_2_/CNFs successfully synthesized by electrospinning combined with the hydrothermal method boast an optimal morphology, with MoS_2_ predominantly distributed on the surface of the fibers.

**Figure 5:**
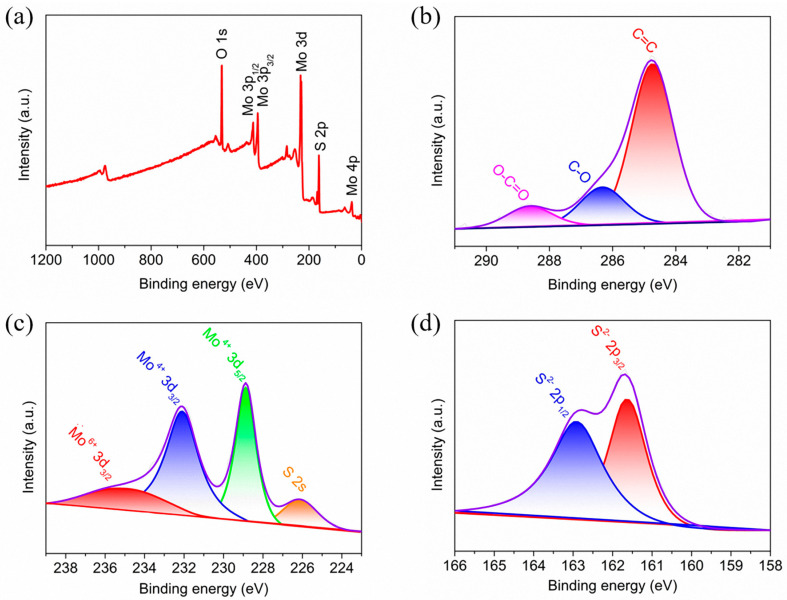
XPS spectra of (**a**) survey spectrum; (**b**) C 1s; (**c**) Mo 3d; (**d**) S 2p of MoS_2_/CNFs.

An X-ray photoelectron spectroscopy (XPS) spectrum for MoS_2_/CNFs composites was conducted to investigate the surface elemental composition and chemical state. Overall, the XPS full spectrum of MoS_2_/CNFs composites showed the coexistence of Mo, C, and S elements, further supporting the composition of the composite consisting of these three elements (Figure 5a). Figure 5b shows a high-resolution C 1s spectrum consisting of three peaks, including the main C=C peak at 284.7 eV and two other peaks at 286.4 and 288.6 eV, corresponding to C-O and O-C=O, respectively. For the XPS high-resolution spectrum of Mo 3d (Figure 5c), two peaks located at 231.9 and 228.8 eV can be attributed to Mo 3d_3/2_ and Mo 3d_5/2_ of Mo^4+^ in MoS_2_ [32,33]. In addition, the peak located at 235.8 eV corresponds to a satellite peak of Mo^6+^, while another peak located at 226.2 eV is attributed to S 2s [34]. Figure 5d shows a high-resolution S 2p spectrum, where the peaks located at 163.1 and 161.9 eV belong to S 2p_1/2_ and S 2p_3/2_ [35,36]. Therefore, MoS_2_ and CNFs are bound through C-O-Mo bonds, indicating a strong interaction between Mo species and functional groups present on CNFs, which originate from the peroxide and carbonization processes of PAN-[37].

**Figure 6:**
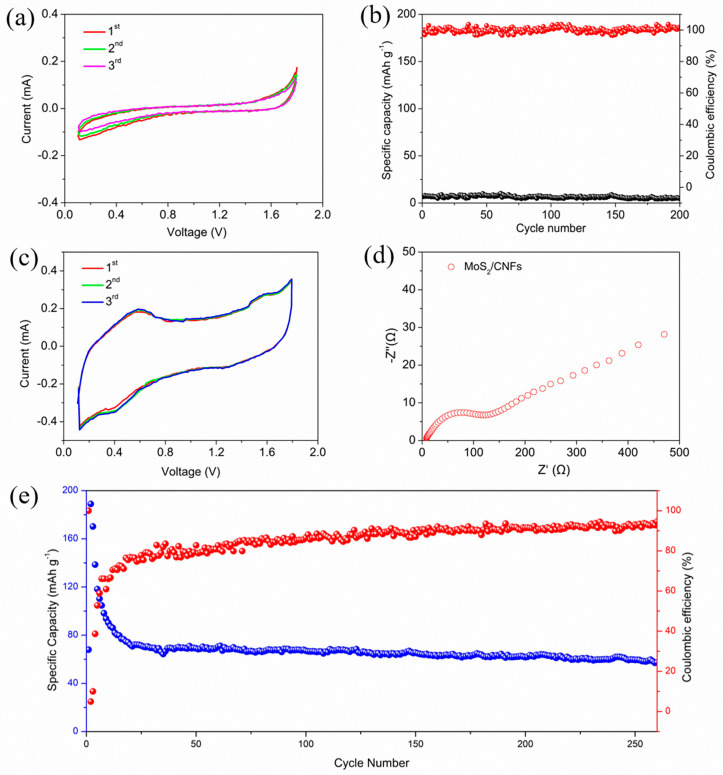
CV curves of: (**a**) CNFs, (**c**) MoS_2_/CNFs; cycling performance at 100 mA g^−1^ of (**b**) CNFs, (**e**) MoS_2_/CNFs; (**d**) Nyquist plots of MoS_2_/CNFs.

Figure 6 shows the electrochemical performance of soft-pack batteries assembled with MoS_2_/CNFs and CNFs as the electrodes. Since the MoS_2_/CNFs electrodes tested in the experiment are composite materials, it is necessary to analyze the carbon substrate to understand its role in the composite electrode. Figure 6a depicts the CV curve of pure CNFs as the electrode. It is evident that the battery exhibits no electrochemical activity at this voltage window, indicating that the carbon material only plays a role as a self-supporting substrate for MoS_2_. Furthermore, in comparison to its corresponding constant current charge–discharge curve, it can be clearly seen that the specific capacity released by the electrode at a current density of 100 mA g^−1^ is almost negligible (Figure 6b). Consequently, it can be inferred that using carbon nanofibers as the matrix to support the active substance has no impact on the electrochemical performance of the battery when operated under a voltage range of 0.1–1.8 V.

Figure 6c displays the CV curve of the MoS_2_/CNFs composite material serving as the cathode of AIBs. In contrast to the pure CNFs cathode, the battery demonstrates electrochemical activity within this voltage window, showcasing a subtle oxidation reduction peak. For the cathodic sweep peak, the oxidation peak is located at approximately 0.55 V and 1.58 V, while for the anodic sweep peak, the reduction peak positions of the curve are approximately 1.23 V and 0.42 V. Therefore, utilizing MoS_2_ as the active substance in AIBs manifests battery characteristics, indicating the potential for cyclic performance. The Nyquist plot is shown in Figure 6d, revealing the kinetic characteristics of the MoS_2_/CNFs electrode. It can be clearly observed that the impedance spectrum of the electrode consists of a semicircle and a slope, where the semicircle is related to the charge transfer dynamics between the electrode interface and the electrolyte, and the slope indicates the diffusion resistance of ions. The Nyquist curves are simulated by the modified Randles equivalent circuit (Figure S3, Supporting Information). From the fitted curve, it can be seen that the resistance value of the electrode is relatively small (128 Ω), indicating the high charge/ion conductivity of the electrode. After cycling, the hybrid electrode exhibited significantly enhanced charge transfer kinetics with a remarkably reduced charge transfer resistance of 66 Ω compared to the fresh cell (Figure S2, Supporting Information) [38]. The main reason is that the 3D network structure of the composite material is conducive to electrolyte penetration into the electrode, and the continuity of CNFs shortens the electron migration path. As illustrated in Figure 6e, since pure CNFs lack electrochemical activity within this voltage window, the curve reflects the capacity released by MoS_2_. The initial capacity of the battery is approximately 180 mAh g^−1^, but it experiences rapid capacity degradation in the initial cycles. After 30 cycles, the battery gradually tends to a stable state, reaching a capacity of about 60 mAh g^−1^. In subsequent cycles, the battery displays good stability, and after 260 cycles of charging and discharging, the AIBs possess a capacity of approximately 53 mAh g^−1^. To evaluate the rate performance of the MoS_2_/CNFs electodes, charge/discharge cycles at different current densities from 100 to 500 mA g^−1^ were performed, as shown in Figure S4 (Supporting Information). Furthermore, we have compared the performance of MoS_2_/CNFs for application in AIBs with several other transition metal dichalcogenides in Table S1 (Supporting Information).

**Figure 7:**
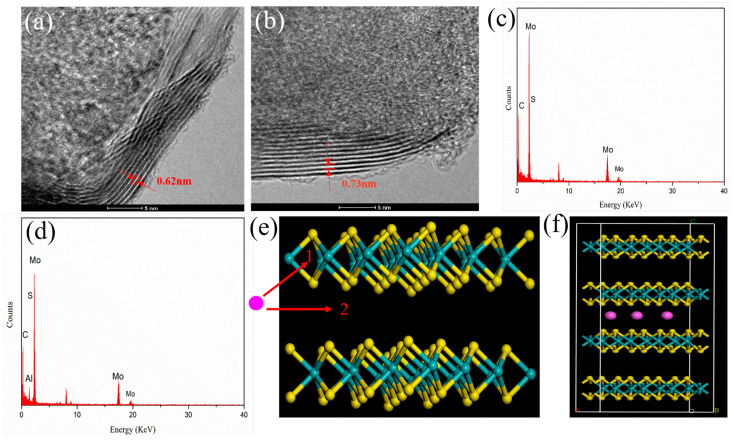
(**a**,**b**) HR-TEM images of the MoS_2_/CNFs before and after cycled; (**c**,**d**) the EDS spectrum of the MoS_2_/CNFs before and after cycled; (**e**) the crystal structure of MoS_2_ and the possible entry methods of Al^3+^; (**f**) schematic diagram of the structure of Al^3+^ in MoS_2_ crystals. The red, green, and yellow circles represent Mo, Al, and S atoms.

MoS_2_, serving as a host material, finds extensive applications in various metal secondary batteries (such as Li, Zn, Na, etc.). Its energy storage mechanism is mostly attributed to the deintercalation behavior of metal ions within MoS_2_ crystals. However, AIBs have multiple ions during the charging and discharging processes, including Al^3+^, [Al_2_Cl_7_]^−^ and [AlCl_4_]^−^, among others. To further ascertain which ion undergoes the deintercalation behavior within MoS_2_ crystals, in-depth studies were conducted on the energy storage mechanism of MoS_2_/CNFs electrodes. Figure 7a,b present high-resolution TEM images of MoS_2_/CNFs before and after cycling, respectively. For the pristine MoS_2_/CNFs, the lattice spacing of MoS_2_ is 0.62 nm, which aligns with its (002) crystal plane spacing (Figure 7a). However, as depicted in Figure 7b, the crystal plane spacing of the cycled electrode increased to 0.73 nm, indicating the presence of ion insertion in the MoS_2_ crystal after cycling. The stability of the electrode was assessed by conducting SEM image analysis on the electrode post-cycling, and the obtained results are presented in Figure S5 (Supporting Information). To gain a more intuitive understanding of the composition and structure of the electrode material interface before and after cycling, EDS was also conducted. Upon EDS analysis of the pre- and post-cycling electrodes, it is evident that a distinct aluminum characteristic peak emerges in the EDS spectrum of the post-cycling electrode (Figure 7c,d). Therefore, it can be concluded that aluminum ions undergo a deintercalation process during the charge and discharge cycles, and this behavior can be summarized using the following equation:

$$Cathode:{Al}^{3+}+MoS_{2}+3xe^{-}\overset{discharge/charge}{↔}{Al}_{x}MoS_{2}$$

$$Anode:7\left[Al\right.{\left.{Cl}_{4}\right]}^{-}+Al\overset{discharge/charge}{↔}4\left[{Al}_{2}\right.{\left.{Cl}_{7}\right]}^{-}+3e^{-}$$

However, due to the unique sandwich structure of MoS_2_, molybdenum atoms and sulfur atoms form the spatial structure of S-Mo-S, and each adjacent MoS_2_ layer is separated by van der Waals forces. To further investigate the deintercalation positions of aluminum ions, the experiment incorporated first-principles calculations to predict the potential entry pathways for aluminum ions. Figure 7e illustrates the crystal structure of MoS_2_ and the possible entry pathways for aluminum ions. Initially, it was hypothesized that aluminum ions could be deintercalated in both channel 1 and channel 2 in MoS_2_. However, the computational results revealed that the energy required for aluminum ions to be deintercalated in channel 1 is significantly high and nearly unattainable, while the energy required for deintercalation in channel 2 is relatively low, which can possibly attributed to the presence of minor van der Waals forces between layers. Based on this, it can be inferred that during the cycling process of the battery, aluminum ions predominantly undergo deintercalation in channel 2 of MoS_2_. The corresponding computational results are shown in Table S2 (Supporting Information).

## 4. Conclusions

This study delves into the energy storage mechanism of AIBs, with a focus on the layered structure of MoS_2_ as the host material. MoS_2_/CNFs composite materials were synthesized through a combination of electrospinning and the hydrothermal method, serving as the self-supporting electrodes for AIBs. The electrode material boasts a 3D network structure, which not only enhances the conductivity of the electrode, but also effectively prevents the aggregation of MoS_2_ during the synthesis process. As self-supporting electrodes, the AIBs exhibit a residual reversible capacity of 53 mAh g^−1^ at 100 mA g^−1^ after 260 cycles. The experimental and computational findings reveal the presence of Al^3+^ intercalation in MoS_2_/CNFs electrodes during the charge and discharge processes. Based on first-principles calculations, it can be concluded that the energy required for Al^3+^ to be inserted in the interlayer gaps formed by S-Mo-S ion bonds is relatively high, indicating that this entry method is almost impossible. Meanwhile, the formation energy for deintercalation between each MoS_2_ layer is relatively small, approximately 0.64 eV. Therefore, during the charging and discharging processes, Al^3+^ mainly undergoes deintercalation between each layer. The insights gained from this research are expected to guide the design and synthesis of next-generation high-capacity and durable AIBs electrodes, laying the foundation for a more sustainable energy future and facilitating the commercial application of AIBs.

## Data Availability

Data are contained within the article.

## Supplementary Materials

The following supporting information can be downloaded at: https://www.mdpi.com/article/10.3390/nano14050442/s1, Figure S1 Scanning electron microscopy (SEM) images of MoS_2_. Figure S2 Nyquist plots of MoS_2_/CNFs after cycling. Figure S3 Equivalent circuit model. Figure S4 Rate performance of MoS_2_/CNFs electrode at different current density. Figure S5 Scanning electron microscopy (SEM) images of MoS_2_/CNFs after cycling. Table S1 Performance comparison of MoS_2_/CNFs with several other transition metal dichalcogenides for application in AIBs. Table S2 The formation energy of aluminum ions embedded in different channels of MoS_2_. References [39,40,41,42,43,44,45,46,47,48,49,50] are cited in the Supplementary Materials.
