# Supplementary material for: Insight into the Storage Mechanism of Sandwich-Like Molybdenum Disulphide/Carbon Nanofibers Composite in Aluminum-Ion Batteries
Source: Nanomaterials (Basel). 2024 Feb 28;14(5):442. doi: 10.3390/nano14050442 (PMC10934325; doi:10.3390/nano14050442)
Supplement: Supplementary file 1 [file nanomaterials-14-00442-s001.zip › nanomaterials-2865955-supplementary.pdf]

Supplementary Information

Insight into the Storage Mechanism of Sandwich-Like Molybdenum Disulphide/Carbon Nanofibers Composite in Aluminum-Ion Batteries

Xiaobing Wang [1], Ruiyuan Zhuang [2,*], Xinyi Liu [1], Mingxuan Hu [1], Panfeng Shen [1], Jintao Luo [1], Jianhong Yang [3] and Jianchun Wu [3,4,*]

[1] School of Advanced Materials Engineering, Jiaxing Nanhu University, Jiaxing 314000, China; 202145575121@jxnhu.edu.cn (X.W.); 202145675122@jxnhu.edu.cn (X.L.); 202145895115@jxnhu.edu.cn (M.H.); 202145905121@jxnhu.edu.cn (P.S.); 202145905123@jxnhu.edu.cn (J.L.)

[2] School of Mechanical and Electrical Engineering, Jiaxing Nanhu University, Jiaxing 314000, China

[3] School of Materials Science and Engineering, Jiangsu University, Zhenjiang 212013, China; jhyang@ujs.edu.cn

[4] Institute of Nuclear Science and Technology, Sichuan University, Chengdu 610064, China

* Correspondence: zhuangryuan@jxnhu.edu.cn (R.Z.); jcwu@ujs.edu.cn (J.W.)

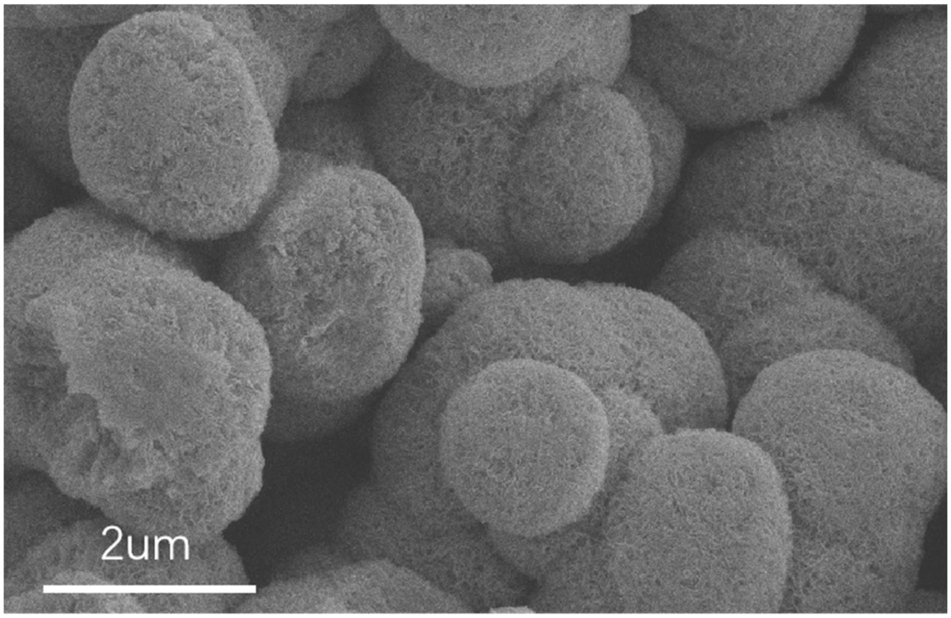

Figure S1 Scanning electron microscopy (SEM) images of MoS_2.

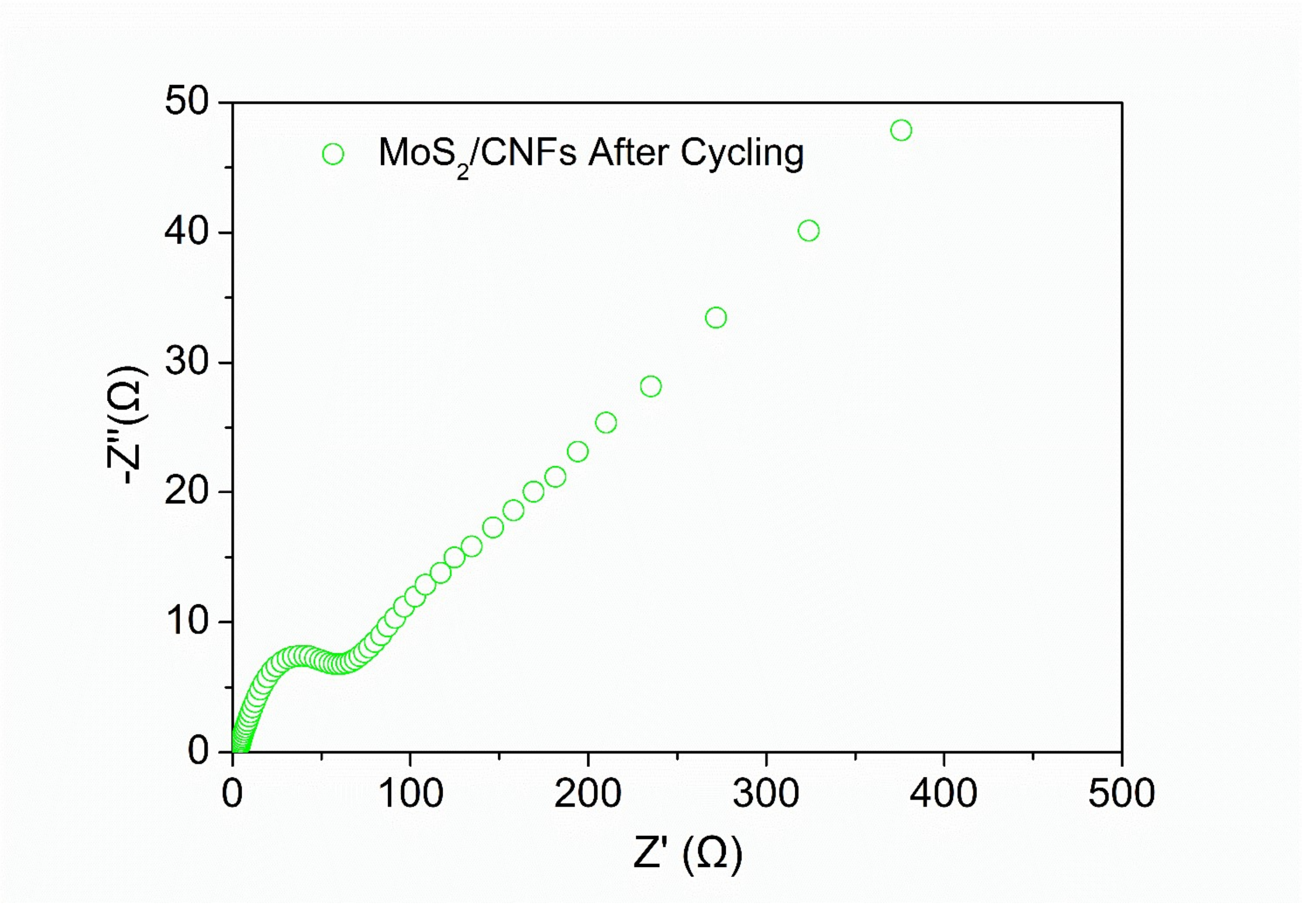

Figure S2 Nyquist plots of MoS_2/CNFs after cycling.

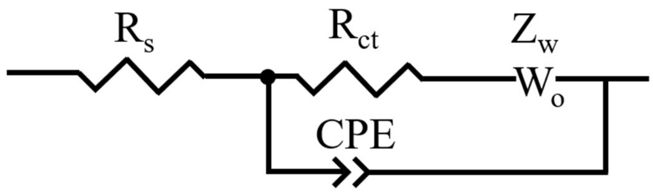

Figure S3 Equivalent circuit model.

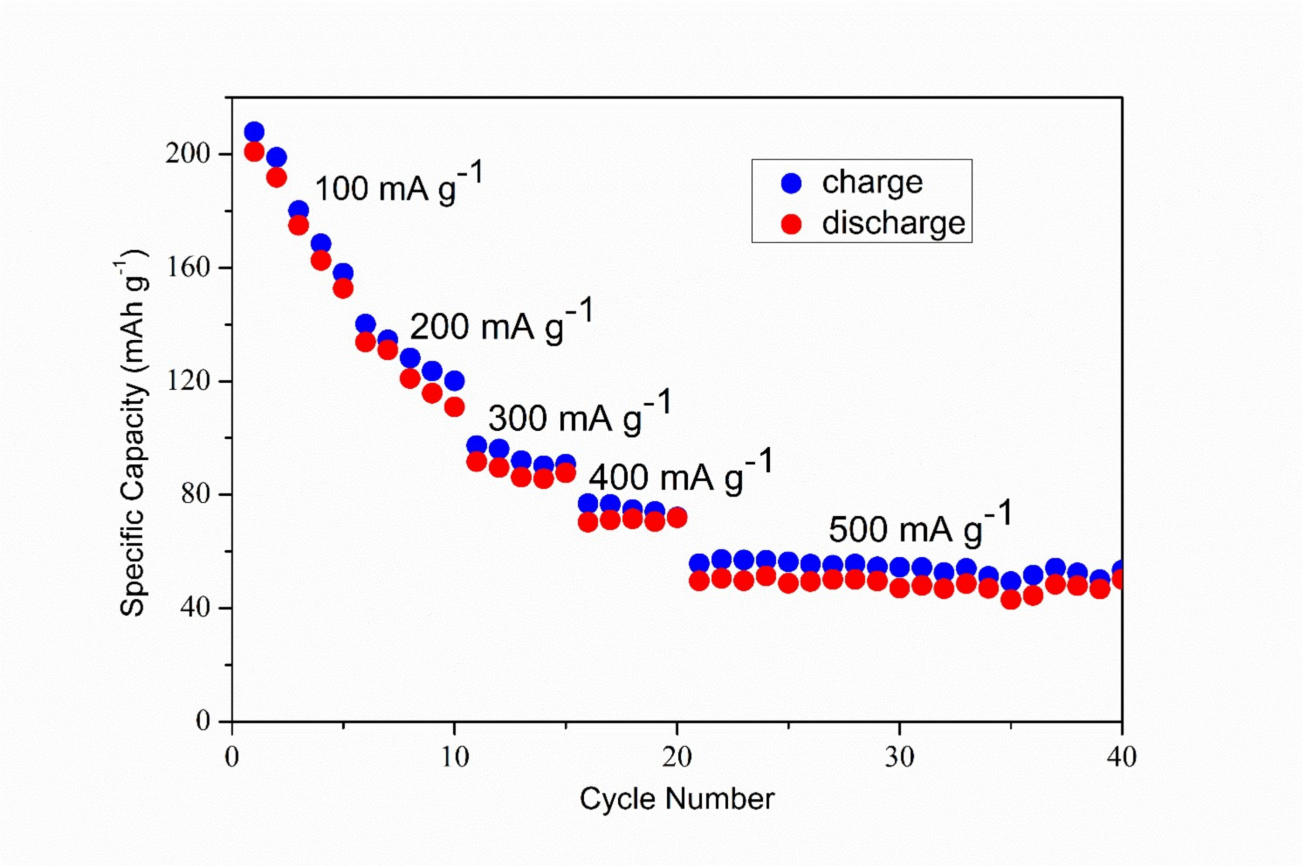

Figure S4 Rate performance of MoS_2/CNFs electrode at different current density.

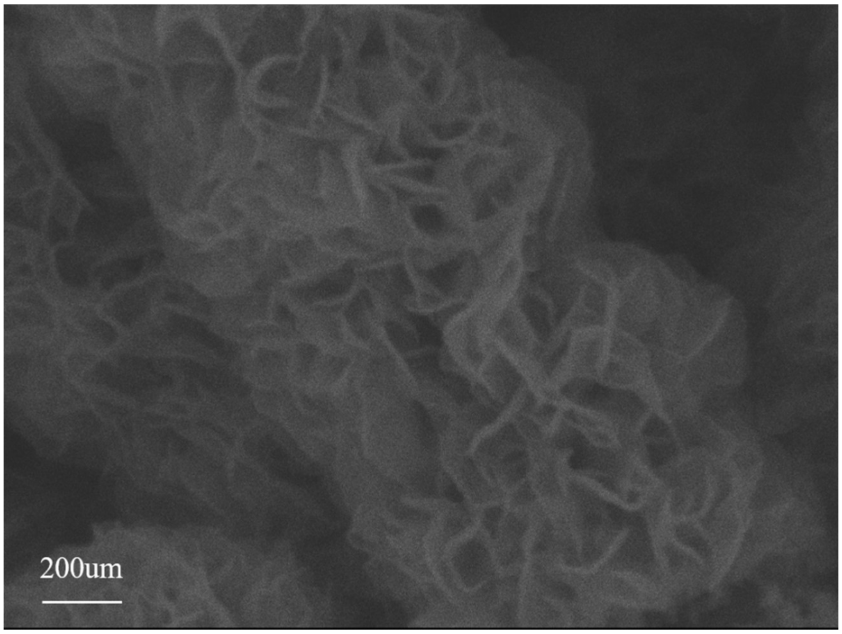

Figure S5 Scanning electron microscopy (SEM) images of MoS_2/CNFs after cycling.

Table S1 Performance comparison of MoS_2/CNFs with several other transition metal dichalcogenides for application in AIBs

Cathode materials	Cycle number	Current density ($mA\ g^{-1}$)	Specific capacity ($mAh\ g^{-1}$)	Ref.
Graphene-VS_2	50	100	50	[39]
VS_4	120	400	129.24	[40]
Ni_3S_2@graphene	100	100	60	[41]
Mo_6S_8	50	12	70	[42]
Co_3S_4 microsphere	150	50	90	[43]
SnS porous film	100	200	70	[44]
Layered TiS_2	50	5	85	[45]
VS_4@rGO	100	300	80	[46]
WS_2@NCNFs	100	100	198	[47]
2D WS_2	500	1000	119	[48]
Graphene-SnS_2	100	200	70	[49]
MoS_2	100	40	66.7	[27]
CoS_2/CNT	100	100	60	[50]
**MoS_2/CNFs**	**200**	**100**	**66**	**This work**

Table S2 The formation energy of aluminum ions embedded in different channels of MoS_2

the possible entry pathways for aluminum ions	Total energy （eV）	Formation energy （eV）
Channel 1	-716.86	5.34
Channel 2	-720.62	0.64
